# The adventive genus *Xantholinus* Dejean (Coleoptera, Staphylinidae, Staphylininae  in North America: new records and a synthesis of distributional data

**DOI:** 10.3897/zookeys.65.574

**Published:** 2010-10-29

**Authors:** Adam J. Brunke, Christopher G. Majka

**Affiliations:** Department of Environmental Biology, University of Guelph, Guelph, ON, Canada, N1G 2W1; Nova Scotia Museum, 1747 Summer St., Halifax, NS, Canada, B3H 3A6

**Keywords:** exotic, Coleoptera, *Xantholinus elegans*, Xantholinini

## Abstract

New distributional and bionomic data are provided for species of the genus *Xantholinus *in North America. *Xantholinus elegans *(Olivier 1795) (= *X. jarrigei *Coiffait 1956)is recorded from North America for the first time, based on specimens collected in Ontario, Canada from 2007-2010. The armature of the internal sac of the aedeagus *in situ *is illustrated to aid in identification. *Xantholinus linearis* (Olivier 1795), known previously from the Maritime Provinces of Canada and the eastern United States, is newly recorded from Ontario. *Xantholinus longiventris *Heer 1839 is still only known from western North America. A key is provided to allow recognition of all three species.

## Introduction

The genus Xantholinus Dejean (Staphylininae: Xantholinini) is a diverse, mainly Palearctic group and contains several species that prefer open, disturbed areas, where they often dominate the staphylinid assemblage (Daccordi and Zanett 1989; Balog and Marko 2007). These habits have likely facilitated the accidental importation and subsequent establishment of Xantholinus species into North America. Smetana (1982) reported Xantholinus linearis (Olivier 1795) from both eastern and western portions of the North America and Xantholinus longiventris (Olivier 1795) only from western regions. Since then, several publications have presented either new provincial and state records, or additional locality data for these two species (Smetana 1988, 1990; Majka and Klimaszewski 2008a; Majka et al. 2008).

Recent collections and surveys in Ontario have resulted in the recognition of one additional species in North America and a range extension for Xantholinus linearis. We here summarize all available data for Xantholinus species in North America, present distributional maps, and provide a key for identification of the species known from the continent.

## Material and methods

The aedeagus of Xantholinus elegans (Olivier 1795) was prepared for examination as in Smetana (1982) and photographed using an imaging system by Visionary Digital. The specimen photograph of Xantholinus elegans was taken with the same system. Maps were created using ARC-GIS and Abode Photoshop software. The institutions (and their abbreviations) from which material was examined are as follows:

ACPEAgriculture and Agri-Food Canada, Prince Edward Island, Canada (Christine Noronha)

CBUCape Breton University, Sydney, Nova Scotia, Canada (David. B. McCorquodale)

CGMCChristopher G. Majka Collection, Halifax, Nova Scotia, Canada (Christopher G. Majka)

DEBUUniversity of Guelph, Guelph, Ontario (Stephen Marshall)

DENHUniversity of New Hampshire, Durham, New Hampshire, USA (Donald Chandler)

DHWCDavid H. Webster Collection, Kentville, Nova Scotia, Canada (David. H. Webster)

NBMNew Brunswick Museum, Saint John, New Brunswick, Canada (Donald McAlpine)

NSMCNova Scotia Museum, Halifax, Nova Scotia, Canada (Christopher G. Majka)

NSNRNova Scotia Department of Natural Resources, Shubenacadie, Nova Scotia, Canada (Jeffrey Ogden)

SMUSaint Mary’s University, Halifax, Nova Scotia, Canada (Doug Stongman)

UMNBUniversité de Moncton, Moncton, New Brunswick, Canada (Pauline Duerr)

## Results

### Xantholinus elegans

(Olivier 1795)

Staphylinus elegans Olivier 1795: 19; as Xantholinus elegans: Smetana in Löbl and Smetana 2004.

#### Materials.

All specimens studied are deposited in DEBU

**CANADA: ONTARIO:** *Peterborough County:* 5 Km SW of Marmora, under fresh horse dung, 31-VII-2010, A. Brunke (1).*Waterloo Region*: Blair, Rare Charitable Research Reserve, near Whistlebare Rd., soybean field, pitfall trap, 27-VII-2010, A. Brunke (1); *Wellington County*: Arkell, Arkell Research Station, under loose sod beside canola field, 20-VII-2007, A. Brunke (1); Eramosa, Eramosa Rd. and Wellington Rd. 29, soil in agricultural field, corn in previous year, 8-VI-2010, A. Brunke (1); Eramosa, Eramosa Rd. and Wellington Rd. 29, soybean field, pitfall trap, 13-VII-2010, A. Brunke (1); Eramosa, Eramosa Rd. and Wellington Rd. 29, soybean field, pitfall trap, 10-VIII-2010, A. Brunke (1); Guelph, Gordon St. and Wellington Ave, on sidewalk near dry field, 23-VIII-2008, A. Brunke and D.K.B. Cheung (1); Guelph, Arboretum, woods edge in leaf litter, 11-IX-2008, M. Bergeron, S. Paeiro and D.K.B. Cheung, (1); Guelph. University of Guelph campus, under rocks, 22-VII-2009, C. Ho and S.P.L. Luk, (2); Guelph, Victoria Rd. and Conservation Line, soybean field, pitfall trap, 4-VIII-2009, A. Brunke, (1). Guelph, Stone Rd., heavily disturbed forest edge under rock, coll. as larva 10-IV-2010, emerged 15-V-2010, A. Brunke (1).

**Figure 1.**
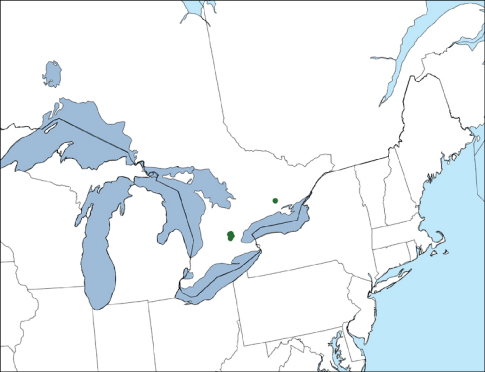
Distribution of Xantholinus elegans in North America.

**Figure 2.**
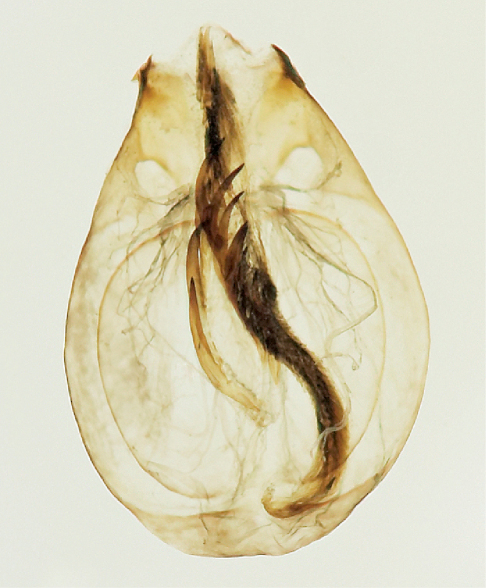
Aedeagus of Xantholinus elegans.

Xantholinus elegans is newly recorded from North America based on the above specimens collected near Guelph and near Marmora, Ontario, Canada (Fig. 1). Dissected specimens key out to Xantholinus jarrigei Coiffait in Coiffait (1972), a species synonymized with Xantholinus elegans (Olivier) by Drugmand (1994).The aedeagus is illustrated in Fig. 2 and those of the other two species were illustrated by Smetana (1982). Most specimens were found in strongly disturbed areas and all individuals were brachypterous. One larva was found under a rock at the edge of a disturbed woodlot in April 10, 2010 and was subsequently reared to an adult on May 15th. The larva was provided with soil from the collection site which included oribatid mites and early-instar Oniscus asellus, although the larva was never observed to feed.

### Xantholinus linearis

(Olivier 1795)

Staphylinus linearis Olivier 1795: 19; as Xantholinus linearis: Smetana in Löbl and Smetana 2004.

#### Materials.

**CANADA: ONTARIO:** *Huron County*: Auburn, 1km NE of Baseline Rd. and Londesboro Rd., wooded hedgerow, pitfall trap, 23-XI-2009, A. Brunke (1); Auburn, Hullett-McKillop Rd. nr. Limekiln Rd., soybean field, pitfall trap, 4-VIII-2010, A. Brunke (1); Goderich, Sharpes Creek Line, wooded hedgerow, pitfall trap, 19-X-2009 (1), 16-XI-2009 (1), A. Brunke. *Waterloo Region*: Blair, Dickie Settlement Rd. nr. Whistlebear Golf Club, pitfall trap, soybean field, 15-XI-2009, A. Brunke (1); wooded hedgerow, 10-XI-2009, (1), 24-XI-2009 (15), A. Brunke; Blair: *rare* charitable research reserve, Fountain St. and Limerick Rd., pitfall trap, soybean field, 15-IX-2009, A. Brunke (1), wooded hedgerow, 27-X-2009 (2), 10-XI-2009 (6), 24-XI-2009 (14), A. Brunke; Blair, nr. Whistlebare Rd., soybean field, pitfall trap, 29-VI-2010 (2), 13-VII-2010 (6), 27-VII-2010 (2), A. Brunke; *Wellington County*: Eramosa, Eramosa Rd. and Wellington Rd. 29, agricultural hedgerow, pitfall trap, 18-V-2010, A. Brunke (1); Guelph, University of Guelph, debris under dead hawk, 27-VI-2008, A. Brunke (1), under patio stone, 2-IV-2009, S.P.L Luk (1), leaf litter in woodlot, 2-IV-2009, A. Brunke (1), on brick wall, 9-XI-2009, S.P.L Luk (1); Guelph, Victoria Rd. and Conservation Line, wooded hedgerow, pitfall trap, 20-X-2009 (2), 17-XI-2009 (2), A. Brunke.

**Figure 3.**
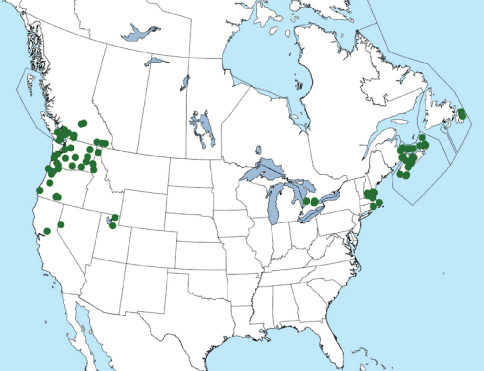
Distribution of Xantholinus linearis in North America. Distribution incorporates previous records from the literature (Smetana 1982, 1988, 1990; Sikes 2003, Majka and Klimaszewski 2008a).

Xantholinus linearis is newly recorded from Ontario based on numerous recent collections from the southern portion of the province. Specimens were collected mainly in agricultural or urban settings in open or forest edge habitat. The earliest Canadian records are from 1949 (in Nova Scotia and Newfoundland) and the earliest North American ones are from 1930 (in Washington state) (Majka and Klimaszewski 2008a). The current distribution of Xantholinus linearis is summarized in Fig. 3.

### Xantholinus longiventris

Heer 1839

Xantholinus longiventris Heer 1839: 247; Xantholinus longiventris: Smetana in Löbl and Smetana 2004.

#### Distribution.

**Figure 4.**
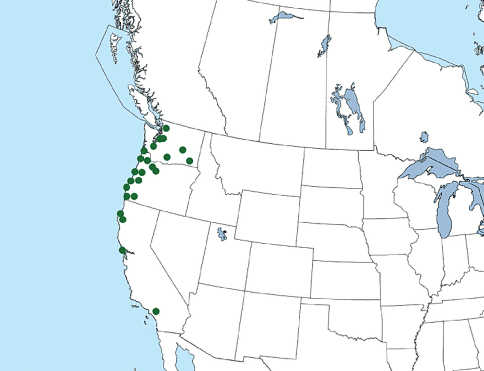
Distribution of Xantholinus longiventris in North America. Distribution incorporates previous records from the literature (Smetana 1982, 1988).

The current distribution of Xantholinus longiventris is summarized in Fig 4.

#### Key to the Xantholinus species of North America

**Table:** 

1	Body distinctly bicolored: head black, sharply contrasting with red-orange pronotum and elytra (Fig. 5, 6)	Xantholinus elegans
1’	Body not distinctly bicolored: body entirely medium to very dark brown, with the elytra often slightly paler (Fig. 7)	2
2	Pronotum with distinct microsculpture of transverse waves present on most of pronotum; occurring in eastern and western North America	Xantholinus linearis
2’	Pronotum with, at most, fragments of microsculpture on the anterior angles; known only from western North America	Xantholinus longiventris

**Figure 5.**
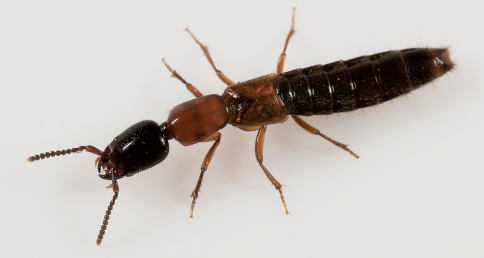
*In vivo* habitus of Xantholinus elegans, from Guelph, Ontario, Canada. Photo by D.K.B. Cheung.

**Figure 6.**
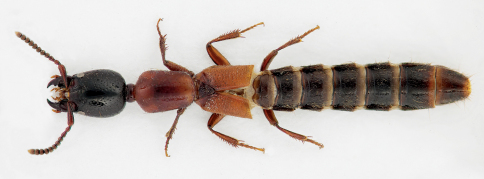
Dorsal habitus of Xantholinus elegans.

**Figure 7.**
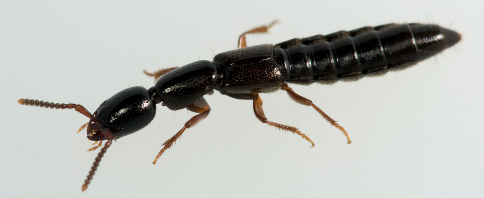
*In vivo* habitus of Xantholinus linearis, from Guelph, Ontario, Canada. Photo by Stephen Marshall.

## Discussion

Xantholinus elegans is certainly a recent accidental introduction to North America as it was not included in Smetana (1990), and the earliest specimen known is from 2007. In its native range, Xantholinus elegans is distributed widely in the western Palearctic region and recorded from Austria, Belgium, Bosnia Herzegovina, Czech Republic, France, Great Britain, Germany, Hungary, Ireland, Italy, Luxembourg, The Netherlands, Poland, Slovakia and Spain (Smetana in Löbl and Smetana 2004). In Europe, it prefers sandy soils and is a bivoltine species with most adults collected in spring and late summer (Daccordi and Zanetti 1989; Drugmand 1994). While most North American specimens were found on sandy soil, adults were collected throughout the summer and sparingly in spring and fall. Further collecting should help determine whether this is a collecting artefact or a shift in seasonality in response to a different geographic area. The majority of specimens have been collected in disturbed habitats and it is unknown if this species will invade habitats with little to no recent human disturbance. It is unclear whether the easternmost record (Marmora) (Fig. 1) represents an isolated population as a result of human-aided dispersal, or if it indicates an inadequately sampled, broader distribution in southern Ontario.

The method of introduction is unknown but may be related to the importation of plant stock or associated materials as Xantholinus linearis was intercepted twice in soil with primrose and moss shipments from Europe in the 1930’s (Smetana 1982). Other predatory beetles are suspected to have become established via plant stock importation (Spence and Spence 1988). Although exotic staphylinids are typically considered to enter eastern North America via Atlantic Canadian introduction points in Nova Scotia, Newfoundland, and in Massachusetts – many associated with historic shipments of dry ballast material (Smetana 1995; Majka and Klimaszewski 2008b) – examination of recent material from the University of New Hampshire Insect Collection and numerous collections in Maritime Canada have not turned up specimens of this species. It appears that the North American occurrence of Xantholinus elegans represents an inland introduction, similar to that of the Emerald Ash Borer, which was first detected in Michigan/southern Ontario in 2002 (Poland et al. 2006). Although Xantholinus elegans is a brachypterous species (Assing 1993; Drugmand 1994) and is unable to disperse aerially, other beetles introduced to North America were found to disperse readily, despite their brachyptery (Spence and Spence 1988). The availability of suitable, open habitat in eastern North America may provide for the expansion of its range to include regions other than Ontario.

Xantholinus linearis was considered to be well-established in both eastern and western North America by Smetana (1982, 1988, 1990) and data presented in this paper demonstrate that it is continuing to expand its range towards the centre of the continent. This species was previously known from British Columbia, Nova Scotia, New Brunswick, Newfoundland and Prince Edward Island in Canada, and California, Idaho, Massachusetts, New Hampshire, Nevada, Oregon, Rhode Island, Utah, and Washington in the United States (Smetana 1982; Smetana 1990; Chandler 2001; Sikes 2003; Majka and Klimaszewski 2008a; Klimaszewski et al. 2010). While it has been known from Atlantic Canada since 1949, it appears to have only recently reached Ontario, as it is missing from collections made prior to 2008. Specimens from Pennsylvania and New York were clearly stated as ‘interceptions’ by Smetana (1982) and should not be considered as evidence that this species occurs there. Interestingly, recent surveys of open field habitat in both these states have not detected Xantholinus linearis (Byers et al., 2000). Further survey work is needed to fully delimit the eastern range of this species.

Xanthlinus longiventris is still known only from the western United States (California, Oregon, Washington) and has not been reported from additional states or any provinces since it was treated in Smetana (1982). In North America, habitat data from specimens suggests that Xantholinus longiventris, while it often co-occurs with Xantholinus linearis, prefers a higher level of moisture (in moss, near water etc.) as it has not been collected from drier urbanized places where the latter species is often found. This species’ range in North America is probably confined by the Rocky Mountain system and will likely remain stable in the absence of secondary introductions.

Three species of Xantholinus are now known to have established themselves in North America via human activity. Of these, at least Xantholinus linearis is apparently continuing to expand its distribution towards the centre of the continent and may be detected in additional provinces and states in the future. This paper provides a current synthesis of distributional information and facilitates the identification of a previously unrecognized species for the North American fauna. A complete review and identification manual for the entire Xantholinini in eastern North America is currently in preparation.

## Supplementary Material

XML Treatment for Xantholinus elegans

XML Treatment for Xantholinus linearis

XML Treatment for Xantholinus longiventris
